# Group G Streptococcal Bacteremia in Jerusalem

**DOI:** 10.3201/eid1008.030840

**Published:** 2004-08

**Authors:** Ronit Cohen-Poradosu, Joseph Jaffe, David Lavi, Sigal Grisariu-Greenzaid, Ran Nir-Paz, Lea Valinsky, Mary Dan-Goor, Colin Block, Bernard Beall, Allon E. Moses

**Affiliations:** * Hadassah-Hebrew University Medical Center, Jerusalem, Israel;; † Ministry of Health Central Laboratory, Jerusalem, Israel;; ‡ Hebrew University-Hadassah Medical School, Jerusalem, Israel;; ¶ Centers for Disease Control and Prevention, Atlanta, Georgia, USA

**Keywords:** Streptococcus group G, emm typing, epidemiology, recurrent bacteremia

## Abstract

Group G *Streptococcus* (GGS) can cause severe infections, including bacteremia. These organisms often express a surface protein homologous to the *Streptococcus pyogenes* M protein. We retrospectively studied the characteristics of patients from the Hadassah Medical Center with GGS bacteremia from 1989 to 2000. Ninety-four cases of GGS bacteremia were identified in 84 patients. The median age was 62 years, 54% were males, and 92% had underlying diseases (35% had a malignancy, and 35% had diabetes mellitus). The most frequent source for bacteremia was cellulitis (61%). *emm* typing of 56 available isolates disclosed 13 different types, including 2 novel types. Six patients had recurrent bacteremia with two to four bacteremic episodes, five had chronic lymphatic disorders, and two had *emm* type *stG*840.0 in every episode. Recurrent bacteremia has not been described for invasive group A *Streptococcus*. We describe an entity of recurrent GGS bacteremia, which is associated with lymphatic disorders and possibly with *emm stG*840.0.

## Abstract 2

Recurrent group G *Steptococcus* bacteremia, associated with lymphatic disorders and possibly *emm* *stG*840.0, is described.

Large colony-forming group G β-hemolytic streptococci (GGS) were first isolated in patients with puerperal sepsis in 1935 (1). GGS are known to be commensals and pathogens in domestic animals. In humans, they may colonize the pharynx, skin, gastrointestinal and female genital tract (2). In recent years, GGS have been reported with increasing frequency as the cause of a variety of human infections, such as pharyngitis, cellulitis, meningitis, endocarditis, and sepsis (2–8). Bacteremia attributable to GGS has been related to underlying conditions, such as alcoholism, diabetes mellitus, malignancy, intravenous substance abuse, or breakdown of the skin (2–7,9,10).

The taxonomy of these organisms has been reevaluated in recent years. The Lancefield group G carbohydrate may be encountered in several β-hemolytic streptococcal species, including *Streptococcus anginosus* and *S. canis*, but mainly in *S. dysgalactiae* subsp. *equisimilis*, which is the subject of our study (11). This subspecies also hosts variants with Lancefield group A, C, and L carbohydrates. The subspecies epithet was determined by gene sequencing of the group C species previously named *Streptococcus equisimilis*, which showed it to be indistinguishable from group G *S. dysgalactiae*. This finding resulted in the description of a new taxon, *S. dysgalactiae* subsp. *equisimilis*. The true proportions of the non-G carbohydrates among members of this taxon are difficult to estimate. In a prospective, population-based study of invasive *S. pyogenes* infections (12), we found six isolates with the group A antigen to be *S. dysgalactiae* subsp. *equisimilis*. Among isolates from infections attributable to *S. dysgalactiae* subsp. *equisimilis*, the group C and G antigen was found much more commonly in human infections than group A (GAS) (11).

*S. dysgalactiae* subsp. *equisimilis* and *S. pyogenes* share virulence factors such as streptokinase, C5a peptidase, M protein, streptolysin S, and certain exotoxin genes (13–18). The M protein is an important virulence factor because it confers resistance to phagocytosis (19). M proteins of GAS and GGS obtained from human infections have similar biologic, immunochemical, and structural features (20). The substantial polymorphism exhibited by GAS M proteins has also been described for GGS (21). We have conducted a 12-year retrospective study to establish the incidence, clinical features, epidemiologic characteristics, and *emm* typing of GGS isolates that cause bacteremia in a large tertiary-care center in Jerusalem, Israel.

## Materials and Methods

A retrospective study was conducted from 1989 through 2000 at the Hadassah Hospitals, Jerusalem, Israel. This is a 1,000-bed, tertiary-care center with all major disciplines represented, including hematology, oncology, and bone marrow transplantation. The mean number of annual admissions during the study period was 62,433.

### Clinical Characteristics

We reviewed the records of all patients in whom a positive blood culture of GGS was reported. Demographic and clinical data were collected. Death rates were measured only during hospitalization. Descriptive statistics were performed with the SPSS (SPSS Inc., Chicago, IL) statistical package release 11.01. The Fisher exact test was used for differences in proportions. A two-sided p value of <0.05 was considered significant.

### Microbiologic Methods

Patients with GGS bacteremia were retrieved from the microbiology laboratory database of bacteremia. No more than one isolate per admission was included. Clinical specimens were collected and handled according to standard protocols. During the study period, the BACTEC 460 radiometric system and the BacTAlert (Organon Teknika, Belgium) blood culture system were used.

All catalase-negative, chain-forming, gram-positive cocci that were β-hemolytic on 5% sheep blood agar were Lancefield serogrouped by using kits according to the manufacturer’s instructions (PathDox Strep Grouping, DPC Diagnostic Products Corporation, Los Angeles, CA). We had 56 isolates available for further analysis. These had been stored at –70°C in the laboratory collection of blood culture isolates. All isolates displayed large colonies and did not belong to the *S. anginosus* group ("*S. milleri*").

### Molecular Methods

An overnight growth was resuspended in saline and heated at 70°C for 15 min. Bacteria were then resuspended in 50 µL of 10 mmol/L Tris, 1 mmol/L EDTA, pH 8. Ten microliters of mutanolysin (3,000 U/mL) and 2 µL hyaluronidase (30 mg/mL) were added. After incubation at 37°C for 30 min and heat inactivation at 100°C for 10 min, the supernatant was subjected to polymerase chain reaction (PCR). Fifty-six GGS isolates were *emm* typed. PCR was performed as described (http://www.cdc.gov/ncidod/biotech/strep/doc.htm). Primers used for amplification of GGS DNA were G1F and G1R, previously described by Schnitzler et al. (21).

According to recommendations (http://www.cdc.gov/ncidod/biotech/strep/doc.htm), the sequence of the sense strand of the *emm* hypervariable coding region was determined by using primer 1 (5´ TATTCGCTTAGAAAATTAA 3´) by automated sequencing (Hy Laboratories Ltd., Rehovot, Israel). The sequence of base pair numbers 30–260 was submitted by using the Streptococcal Group A Subtyping Request Form, to the Blast 2.0 Core Facility (http://www.cdc.gov/ncidod/biotech/strep/strepblast.htm), where *emm* type was determined (22).

The 12 strains from recurrent infection were analyzed by pulsed-field electrophoresis (PFGE). Chromosomal DNA was digested with *Sma*I and prepared and analyzed as described with minor modifications (23).

## Results

**Figure 1:**
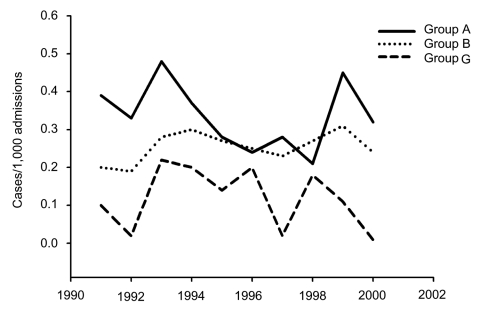
Trends in β-hemolytic streptococcal bacteremia at the Hadassah Medical Center.

The 504 bloodstream isolates of β-hemolytic *Streptococcus* from 1989 through 2000 included the following: 232 (46%) group A, 171 (34%) group B (GBS), 94 (19%) group G, and 7 (1.4%) groups F and C. The 94 episodes of GGS bacteremia involved 84 patients, 6 of whom had recurrent infections. Patient characteristics are summarized in Tables 1 and Table 2. The annual incidence of GGS bacteremia was 0.0–0.2 cases/1,000 admissions during the 12-year study; the incidence ranges for GAS and GBS were 0.2–0.48 and 0.19–0.3, respectively (Figure 1).

**Table 1:** Summary of clinical characteristic of 94 patients with group G streptococcal bacteremia

Characteristic	No. of patients (%)
Age (y)	
<10	3 (3.1)
10–50	28 (29.8)
51–75	42 (44.7)
>75	21 (22.3)
Median (range)	62 (2–92)
Male	51 (54.2)
Median LOH^a^ in days	10
Underlying disorder	
Diabetes mellitus	33 (35.1)
Malignancy	33 (35.1)
Hypertension	18 (19.1)
No disease	8 (8.5)
Type of infection	
Cellulitis	56 (59.6)
Primary bacteremia	18 (19.1)
Soft-tissue infection^b^	4 (4.3)
Bone and joint	4 (4.3)
Endocarditis	3 (3.1)
Respiratory	3 (3.1)
Postpartum	1 (1.1)
Line sepsis	1 (1.1)
Unknown	4 (4.3)
Death rate	5 (5.3)

^a^LOH, length of hospitalization. ^b^Soft tissue infection, pressure sore attributable to diabetes.

**Table 2:** *emm* types of 94 patients with group G streptococcal bacteremia

*emm* types	No. of isolates
*stG*485.0	10
*stG*840.0	7
*stG*6.1	7
*stG*166b.0	6
*stG*4222.0	5
*stG*10.0	3
*stG*5420.0	3
*stC*74a.0	3
*stG*245.0	3
*stG*480.0	3
*stC*36.0	2
*stG*6792.0^a^	2
*stG*6.0	1
*stG*507.1^a^	1

^a^New *emm* types.

Five patients died, but only two of the deaths were directly attributed to the GGS bacteremia. The other three deaths were related to malignancy in two patients and congestive heart failure in one.

Of the 56 GGS isolates available for *emm* typing, we found 13 *emm* types (including 2 subtypes of *stG*6: *stG*6.0 and *stG*6.1). These types included *stG*507.1, a variant of *emmLG*507.0 (GenBank accession no. X79527) and *stG*6792.0 (identical to a partial gene sequence, accession no. AF485842, listed from a blood isolate of *S. dysgalactiae* subsp. *equisimilis*), which were not in the database. The patient with the *stG*507.1 bacteremia had cellulitis, and two patients had the *stG*6792.0 strain: one was a 17-year-old patient with venous malformations of the leg and pelvis who had GGS sepsis and multiorgan failure, and the other was a 92-year-old man with diabetes and cellulitis. No association was found between *emm* type and year of study, season, source of infection, or cellulitis location.

**Figure 2:**
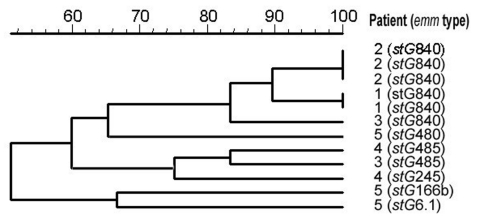
Dendrogram of pulsed-field gel electrophoresis analysis of isolates from patients with recurrent bacteremia. "Patient" refers to numbers from Table 3.

Six patients had recurrent bacteremia, ranging from 2 to 4 episodes per patient (Table 3). All six patients had a community-acquired recurrent cellulitis and were given treatment similar to that received by patients who did not have a recurrent infection. Five patients had chronic lymphatic abnormalities at the infection site compared to 11 of 42 patients with nonrecurrent cellulitis (odds ratio [OR] 14.1, 95% confidence interval [CI] 1.5–134.3, p = 0.012). Two patients had recurrent infection with the same *emm* type, *stG*840.0 (one patient had three episodes, 1 and 7 months apart; the other patient had 2 episodes, 6 months apart). Three patients had a different *emm* type each episode. The isolates of the patient with four recurrent episodes were not available for *emm* typing. PFGE results indicated that isolates recovered from the same patient that shared the same *emm* type were highly genetically related (Figure 2).

**Table 3:** Characteristics of recurrent episodes of GGS bacteremia

Patient	*emm* type				
	Episode 1	Episode 2	Episode 3	Episode 4	Time to recurrence (mo.)
1	*stG*840.0	*stG*840.0			6
2	*stG*840.0	*stG*840.0	*stG*840.0		1, 7
3	*stG*840.0	*stG*485.0			12
4	*stG*485.0	*stG*245.0			21
5	*stG*480.0	*stG*6.1	*stG*166b.0		3, 1
6	Unknown	Unknown	Unknown	Unknown	34, 13, 12

## Discussion

GGS are widely distributed in nature and are recognized as both commensals and pathogens in animals as well as in humans. *S.* *dysgalactiae* subsp. *equisimilis* is part of the normal bacterial flora in humans. *S. dysgalactiae* subsp. e*quisimilis* is the most common species of large colony-forming serogroup G streptococci that is β-hemolytic on sheep blood agar. The animal-associated *S. canis* has rarely been implicated as a human pathogen, although accurate data are not available (11,24). Asymptomatic pharyngeal carriage of GGS has been described in up to 23% of humans (25), and vaginal carriage in 5% of asymptomatic puerperal women (1).

This 12-year retrospective survey identified 94 episodes of GGS bacteremia at the Hadassah-Hebrew University Medical Center. It is one of the two largest series of GGS bacteremia described in the literature to date (26). Some similarities were found in the epidemiologic characteristics of group G streptococcal bacteremia between our study and other studies. Patients were predominantly elderly men. Most patients (92%) had underlying diseases, similar to those (74%–92%) reported previously (2–6,9).

As shown by Auckenthaler et al. (3) and Woo et al. (6), malignancy was one of the most important underlying conditions associated with GGS bacteremia, affecting 35% of our patients. In our patients, the most common malignancy associated with cellulitis was carcinoma of the breast. The most common source of infection among patients with bacteremia with a known source was soft tissue infection, especially cellulitis (61%), 66% of these infections were confined to the lower limbs. Similarly, 48% of our patients with cellulitis had an underlying skin lesion; in 35% of the patients, it was related to lymphatic abnormalities. These abnormalities were mostly attributed to malignancy, surgery, or radiation. Two patients had a congenital lymphatic malformation.

Nineteen percent of our patients had primary bacteremia, which is within the range (11%–52%) described by others (2,3,6,7). The death rate in our study (5%) was lower than that previously reported (8%–30%) (3,6,9). One reason for this finding may be the relatively younger age of our patients: Only 22% of our patients were >75 years of age compared to those from Sylvetsky’s group, where 63% were >75 years of age (9). The small number of patients who eventually died of bacteremia precluded analysis of risk factors associated with death.

In contrast to the previous study from a different hospital in Jerusalem, Israel, describing GGS bacteremia during the same years as our study (9), the yearly incidence of GGS bacteremia did not increase. The dissimilarity in our two groups may be attributable to the differences in ages of the patients and the proportion of men. In addition, the other hospital is a community hospital with a large geriatric department, without neurosurgery or bone-marrow and solid organ transplants.

A noteworthy finding in our series was the high frequency of recurrent GGS bacteremia. We identified six patients with bacteremia, all of whom had recurrent cellulitis. Five had lymphatic drainage abnormalities. Two patients had recurrent bacteremia attributable to the same *emm* type (stG840, patients 1 and 2, Table 3). PFGE results indicated that within each patient the isolates recovered at different time points were clonal (Figure 2) but that the two pairs of *stG*840 isolates had differing PFGE patterns, which suggests that they were different clonal types.

Since two of the patients with recurrent bacteremia had an infection by the same clone, these infections may have been relapses. Recurrence of bacteremia suggests that the initial infection may not provide protective immunity. The question of protective immunity to this bacterium is addressed by Bisno et al. in a murine model of recurrent GGS cellulitis (27). Despite recurrent skin challenge with GGS, the lesion did not decrease in severity, size, or time to heal. Bisno et al. found that, despite the development of demonstrable humoral immune response to M protein, acquired protective immunity did not occur. Possibly GGS downregulates M protein in vivo, thus allowing it to evade these specific antibodies. Immediately initiating antibiotic therapy for the first GGS infection might contribute to the low level of immunity, as has been demonstrated in cases of recurrent GAS tonsillitis (28).

Cellulitis can recur in extremities or other sites where venous and lymphatic circulation has been compromised by processes such as malignancy, lymph node dissection, prior irradiation, trauma, or saphenous venectomy (10,29,30). Nongroup A β-hemolytic streptococci have been implicated as a major cause of cellulitis in the setting of circulatory compromise (31,32). Focusing on the 48 patients with cellulitis, we found that recurrent cellulitis with bacteremia was 14.1 times more likely to develop in patients with lymphatic drainage abnormalities when compared to patients without such abnormalities. Our report is the first to describe the phenomenon of recurrent GGS cellulitis associated with bacteremia in patients with lymphatic abnormalities. Recurrent GGS bacteremia seems to be more common than recurrent GAS bacteremia. In our study of 90 patients with GAS bacteremia (33) who were admitted to the Hadassah Medical Center during a 6-year period, none had a recurrence compared to 6 of 84 patients in this study (p < 0.013). Our patient with four recurrences of GGS bacteremia had in effect five additional episodes, which were not included in our report. Three episodes were cellulitis with GGS bacteremia at other hospitals, and two episodes were severe cellulitis without proven bacteremia. These cases are examples of recurrence of GGS infection in a manner not known to occur with GAS. A recent report suggested that allelic variation of human leukocyte antigen II contributes to the differences in severity of GAS infections (34). The relationship between bacterial factors and host mechanisms of defense in this patient and others with recurrent bacteremia needs further investigation.

To illuminate the unique characteristics of patients with GGS bacteremia, we compared our group of GGS bacteremia patients with two groups of GAS bacteremia patients (33,35). Sex and age of the patients with GGS bacteremia were similar to those with GAS bacteremia in a retrospective study. However, when we compared patients with GAS bacteremia from our hospital participating in a nationwide prospective, population-based study, GGS patients were older and more likely to be men than the GAS patients (35). Thus, the characteristics of the GGS patients may reflect institutional and selection bias attributable to different study methods and may not be a true tendency for older patients.

Serologic M typing was developed years ago for GAS typing, but it has also been used for GGS (36). M protein encoded by *emm* is a virulence factor of GGS similar to the GAS surface protein (20). *emm* typing for both GAS and GGS is based on the heterogeneity of the 5´ ends of the gene, which give rise to different sequence types. More than 120 *emm* types are recognized for GAS, and approximately 40 types of GGS and group C *Streptococcus* (GCS) have been identified (http://www.cdc.gov/ncidod/biotech/strep/emmtypes.htm). Despite the similarities between GAS and GGS, Geyer and Schmidt (37) found that in GCS and GGS, two types of arrangements in the *emm* region differ significantly from the known types of *mga* region in GAS. The conclusion was that Mgc is related to Mga proteins of various types of GAS but forms a distinct cluster.

In previous studies, Lawal et al. used serologic M typing for 103 isolates of GGS. Fifty-six isolates (54%) could be serologically typed into eight serotypes (38). Of 128 isolates, 40 (31%) could be serotyped with six antisera (39). The inability to type a large proportion of GGS by the older serologic method is similar to the situation that exists for GAS (12).

In our 56 GGS isolates available for sequence typing, we found 13 *emm* types. None of our isolates were *emm* nontypeable. Kalia et al. (40) *emm* typed 18 GGS isolates from human infections obtained from various countries. They found 13 *emm* types, the most common of which was *stG*480.0 (3 of 18 isolates compared to 3 of 56 in our study); our most common type was *stG*485.0 (10 of 56 isolates). Thus, *emm* typing provides a useful tool for identifying isolates when compared to traditional M typing.

Tyrrell found a correlation between clusters of GAS M types and patient age (41). We could not find a correlation between *emm* type and clinical features such as patient sex, age, and source of infection or cellulitis location, although our database may not be sufficiently large to draw these correlations.

We found among our GGS isolates *emm* types that were previously described for GCS (*stC*36.0) and for GAS (*stG*245.0). Although *stG*245.0 was originally associated with *S. dysgalactiae* subsp. *equisimilis* harboring group A antigen, it is usually associated with GGS (B. Beall, unpub. data). Kalia et al. described a few GCS *emm* types, which in our study were found in group G streptococci. Certain types are occasionally found in both GGS and GCS (Beall, unpub. data). The dynamics of interspecies transfer of virulence loci between GAS, GGS, and GCS (18,42–45), as well as potential genetic transfer or intragenomic events causing interconversion of group antigen types, remains to be resolved. Our findings that several isolates with *emm* type *stG*840 have different PFGE patterns may support the notion that isolates of GGS with the same *emm* type are not of the same clone. The relationship between GGS *emm* type and clone remains to be examined. In general, GAS *emm* typing, when restricted to the same geographic area and time period, is indicative of clonal type; however Beall et al (46), demonstrated numerous examples of apparently unrelated GAS strains (as judged by independent T agglutination phenotype and opacity factor [*sof*] sequence) sharing the same *emm* sequence type, and, more recently, nonrelatedness between distinct GAS strains of identical *emm* types has been demonstrated through MLST (47).

In summary, we describe 94 cases of GGS bacteremia, observed mainly in older patients with underlying medical conditions. The most frequent portal of entry was the skin. The high rate of recurrence of GGS bacteremia was an unusual and unexpected finding. Clinicians should be alert to this phenomenon, which seems to be more common than recurrent GAS bacteremia. We found that lymphatic drainage disorders were a highly significant risk factor for recurrence, and that *emm* type *stG*840.0 may have a special role in recurring disease.
